# Pharmacogenomic analysis of alarelin acetate-induced hepatotoxicity: a case report and literature review

**DOI:** 10.3389/fmed.2025.1634101

**Published:** 2025-09-29

**Authors:** Fang Yuan, Ping Zhang, Ming Liu, Yuan Li, Bin Xu, Xin Li

**Affiliations:** ^1^Department of Pharmacy, The Third Hospital of Changsha (Changsha Hospital Affiliated to Hunan University), Changsha, Hunan, China; ^2^Hunan Provincial Key Laboratory of Anti-Resistance Microbial Drugs, Changsha, Hunan, China; ^3^Beijing Key Laboratory of Traditional Chinese Medicine Basic Research on Prevention and Treatment for Major Diseases, Experimental Research Center, China Academy of Chinese Medical Sciences, Beijing, China

**Keywords:** alarelin acetate, drug-induced liver injury, pharmacogenomics, human leukocyte antigen, single nucleotide polymorphism

## Abstract

**Background:**

Alarelin acetate, a synthetic gonadotropin-releasing hormone (GnRH) analogue, is widely used to manage endometriosis and hormone-sensitive malignancies. Although its safety profile is generally favorable, we report the first documented case of severe hepatotoxicity associated with alarelin acetate administration.

**Case summary:**

A 37-year-old female participant in a phase I clinical trial developed acute hepatocellular injury following subcutaneous administration of alarelin acetate (150 μg/day). The Roussel Uclaf Causality Assessment Method (RUCAM) yielded a score of 6, indicating a “highly probable” causal relationship between the drug and liver injury. Hepatic enzyme levels normalized within 18 days after drug discontinuation and initiation of hepatoprotective therapy (glycyrrhizin and polyene phosphatidylcholine). Pharmacogenomic profiling identified specific genetic variations that may be associated with alarelin acetate-related hepatotoxicity, including a homozygous *NUDT15* variant (*3/*3 diplotype) and human leukocyte antigen (*HLA*) risk alleles (HLA-DRB1*15:01, HLA-DQB1*06:01).

**Conclusion:**

This novel case highlights the risk of alarelin acetate-related hepatotoxicity. Pharmacogenomic profiling indicated that its hepatotoxicity may be related to gene polymorphisms; however, further research or larger-scale studies are needed to validate these associations.

## Introduction

Alarelin acetate (AA), a synthetic nonapeptide gonadotropin-releasing hormone (GnRH) analogue, induces reversible hypogonadism through pituitary desensitization, making it effective for endometriosis and hormone-dependent cancers (1). While its labeled adverse effects (e.g., vasomotor symptoms and genitourinary atrophy) reflect hypoestrogenic states, hepatotoxicity has not been documented in the current literature.

Drug-induced liver injury (DILI) represents a growing public health concern, with an estimated annual incidence of 23.8/100,000 cases in China, exceeding global rates (2, 3). DILI manifests clinically as acute, subacute, or chronic liver injury, typically characterized by elevated levels of liver function markers, including serum alanine aminotransferase (ALT), aspartate aminotransferase (AST), lactate dehydrogenase (LDH), alkaline phosphatase (ALP), γ-glutamyltransferase (GGT), and total bilirubin (TBil). The diagnostic criteria for DILI include the following: (1) ALT ≥5 × ULN, (2) ALP ≥ 2 × ULN (with concurrent GGT elevation), or (3) ALT ≥3 × ULN and TBil ≥2 × ULN (4). The pathogenesis of DILI is complex, and the known risk factors can be grouped into two categories: drug-related and host-related (5). Emerging evidence implicates genetic polymorphisms in drug metabolism enzymes and human leukocyte antigen (*HLA*) alleles as key determinants of DILI susceptibility.

Pharmacogenomics (PGx) is an interdisciplinary field integrating genetics, genomics, and pharmacogenetics (6). It focuses on the relationship between human genomic information and drug response and uses genomic information to explain the reasons for the differences in the response of different individuals to the same drug (7). Genes associated with drug response include drug-metabolizing enzymes, drug transporters, and specific *HLA* alleles (8). Drugs are typically metabolized in the liver through phase I and/or phase II reactions and are catalyzed by drug-metabolizing enzymes in order to form water-soluble metabolites before excretion.

DILI initiation frequently involves drug metabolism pathways, particularly those mediated by cytochrome P450 enzymes (CYP450) and UDP-glucuronosyltransferases (UGTs) (9). Polymorphisms in genes encoding drug-metabolizing enzymes, such as UGTs, glutathione S-transferases (GSTs), and N-acetyltransferases (NATs), are associated with the risk of DILI (10, 11). The *HLA* complex represents the most polymorphic system in humans, comprising highly conserved major histocompatibility complex (MHC) molecules that are critical for immune function (12). Except for monozygotic twins, *HLA* genotypes are unique to each individual. The *HLA* genes are located on the short arm of chromosome 6 and are classified into class I, II, and III regions based on their gene structure and function (13). *HLA* class I and II molecules primarily present antigens to T cells, while class III genes encode immune-related proteins, such as complement components, cytokines, and heat shock proteins. Experts in drug metabolism and toxicology suggest that *HLA* molecules present drug-derived antigens and are involved in the pathogenesis of DILI (14).

Herein, we report a case of DILI following treatment with alarelin acetate in a previously healthy individual. To the best of our knowledge, this is the first documented case of alarelin acetate-induced DILI. Furthermore, we sought to identify novel rare or low-frequency (minor allele frequency (MAF) < 5%) single-nucleotide polymorphisms (SNPs) that may potentially contributing to susceptibility to DILI associated with AA.

## Participants and methods

### Study design

The study protocol was approved by the Changsha Third Hospital Ethics Committee (NO. LZ-BARL-PK-01) and was registered on ClinicalTrials.gov (NCT2023LP02263). All participants self-identified as Han Chinese and provided written informed consent.

### DILI causality assessment for alarelin acetate

The Roussel Uclaf Causality Assessment Method (RUCAM) was used to determine the causal relationship between liver injury and alarelin acetate (15).

### Pharmacogenomic analysis

Genomic DNA was extracted from peripheral blood using the EasyPure Blood Genomic DNA Kit (TransGen Biotech) and quantified using agarose gel electrophoresis.

Library preparation involved enzymatic fragmentation, end repair, A-tailing, adapter ligation, and PCR amplification. The resulting libraries were then hybridized with custom RNA probes designed to capture genomic regions relevant to PGx studies. Following hybridization, target fragments were enriched using streptavidin-coated magnetic beads. After the quality control assessment, high-throughput sequencing was performed. PGx-relevant variants were identified using GATK (version 3.8) analysis.

The probe panel captured 4,453 PGx-related loci, covering a span of 24.44 kb. This allows for a comprehensive genomic characterization. Sequencing parameters were optimized to ensure that critical loci achieved sufficient coverage, with read depths consistently ranging from 10× to 20×, thereby enhancing data reliability and accuracy.

### *HLA* high-resolution genotyping

Genomic DNA extraction and quantification were performed as described above.

Library preparation involved identical enzymatic fragmentation, end repair, A-tailing, adapter ligation, and PCR amplification. The libraries were hybridized with DNA probes that specifically targeted all exons and critical intronic regions of 22 *HLA* genes (Table 1), covering approximately 300 kb of genomic sequence. Streptavidin-coated magnetic beads were used for target enrichment. The post-enrichment libraries underwent quality control before high-throughput sequencing. *HLA* genotyping was determined through specialized bioinformatics analysis of the sequencing data.

**Table 1:** 22 *HLA* gene.

Type	A, B, C, DRB1, DQA1, DQB1, DPA1, DPB1, DMA, DMB, DOA, DOB, DRA, DRB3, DRB4, DRB5, E, F, G, TAP2, MICA, MICB

Information on the reagents used in the pharmacogenomic analysis and *HLA* high-resolution genotyping experiments is provided in Supplementary Table 1.

## Case report

A 37-year-old woman participated in a phase I clinical trial titled “A single-center, non-randomized, open-label, positive drug-controlled study evaluating the safety, tolerability, pharmacokinetics and pharmacodynamics of a single subcutaneous injection of alanyl acetate in healthy Chinese adult female subjects” at Changsha Third Hospital on 26 April 2024. The participant met the inclusion criteria, had no significant past medical history or drug allergies, no history of substance abuse, and had not used any concomitant medications in the 4 weeks prior to enrollment. No specific negative behaviors (smoking, alcohol use, or recreational drug use) were reported. Laboratory results were within the normal range, and the liver function test (conducted on 27 April 2024) showed the following: ALT 17.2 U/L, AST 21.4 U/L, LDH 153 U/L, and TBil 14.5 μmol/L.

The participant was randomly assigned to the reference group and received alarelin acetate (BBCA Pharmaceutical Co., Ltd., China). Subcutaneous administration (150 μg/day) began on 30 April 2024 and continued for 14 consecutive days. On 1 May 2024, the laboratory test revealed elevated enzyme levels: ALT 432.7 U/L, AST 584.9 U/L, and LDH 1,054.6 U/L; however, the participant remained asymptomatic. A repeat test on 2 May 2024 showed further progression, with ALT 1,117.4 U/L, AST 532.0 U/L, and LDH 303.9 U/L. The investigator concluded that the participant developed significant transaminitis (ALT: 17.2 → 432.7 → 1,117.4 U/L and AST: 21.4 → 584.9 → 532.0 U/L), fulfilling Hy’s Law criteria for severe DILI (ALT ≥ 5 × ULN). Biochemical resolution followed apparent first-order kinetics (estimated ALT t1/2 = 3.2 days) after drug discontinuation. Due to safety concerns, the principal investigator withdrew the participant from the trial.

An immediate hepatology consultation confirmed a diagnosis of DILI. Treatment with compound glycyrrhizin tablets and polyene phosphatidylcholine capsules was initiated. Follow-up liver function tests indicated a progressive improvement in the participant’s condition:

- 3 May 2024: ALT 942.9 U/L and AST 227.5 U/L (significant decrease).
- 7 May 2024: ALT 256.8 U/L and AST 63.5 U/L (further decrease).
- 14 May 2024: ALT 46.1 U/L (near normalization) and AST 20.4 U/L (normal) (Table 2).

**Table 2:** Laboratory parameters of the subject during follow up.

Date	ALT (U/L)	AST (U/L)	LDH (U/L)	ALP (U/L)	GGT (U/L)	TBil (μmol/L)
04.27	17.2	21.4	153	50	11	14.5
05.01	432.7	584.9	1,054.6	74.4	47	8.7
05.02	1,117.4	532	303.9	74	47	8.4
05.02	1,190.5	475.4	280.9	/	41	/
05.03	942.9	227.5	232	75	47	7.6
05.07	256.8	63.5	/	/	/	/
05.14	46.1	20.4	/	/	/	/

ALT, serum alanine aminotransferase; AST, aspartate aminotransferase; LDH, lactate dehydrogenase; ALP, alkaline phosphatase; GGT, γ-Glutamyltransferase; TBil, total bilirubin.

The participant remained asymptomatic throughout the 18-day follow-up period, and the adverse event (AE) resolved.

### PGx and *HLA* gene analyses

**Figure 1:**
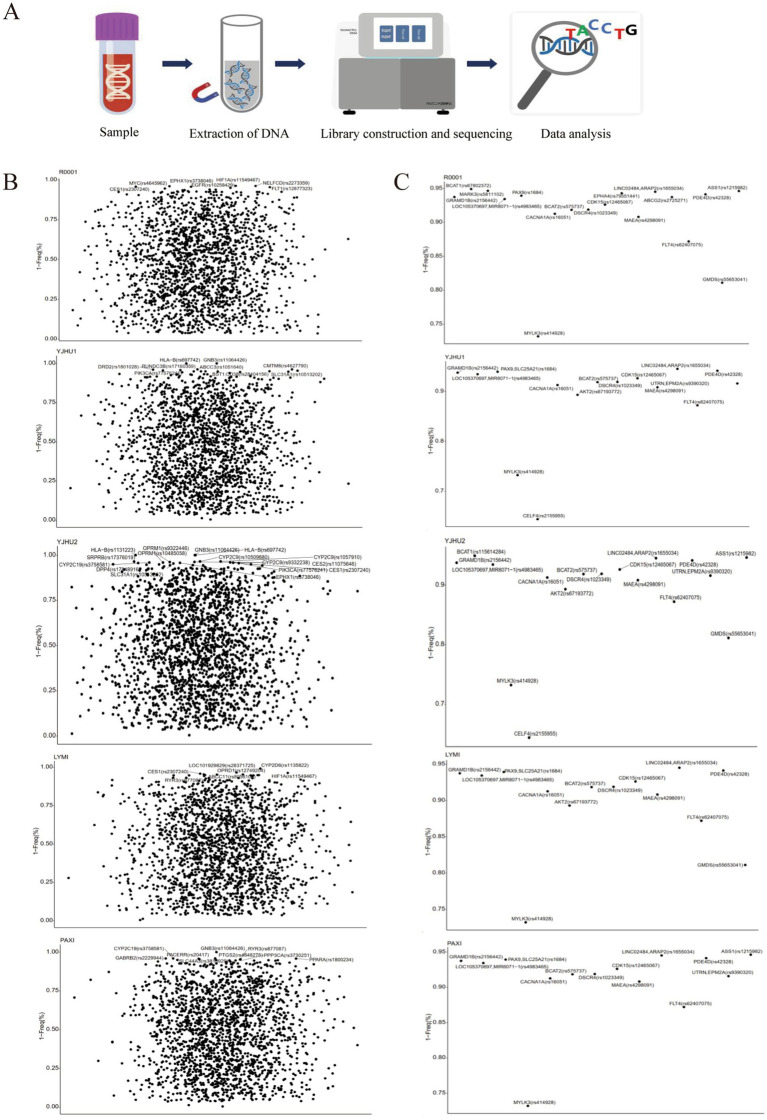
**(A)** Flowchart of the pharmacogenomic analysis and HLA gene analysis. **(B)** The possibility of drug-gene involvement in alarelin acetate-induced liver injury. Loci with a gene frequency of less than 5% are annotated. **(C)** The possibility that alarelin acetate-induced liver injury is associated with highly variable drug-gene loci specific to the Chinese population.

**Figure 2:**
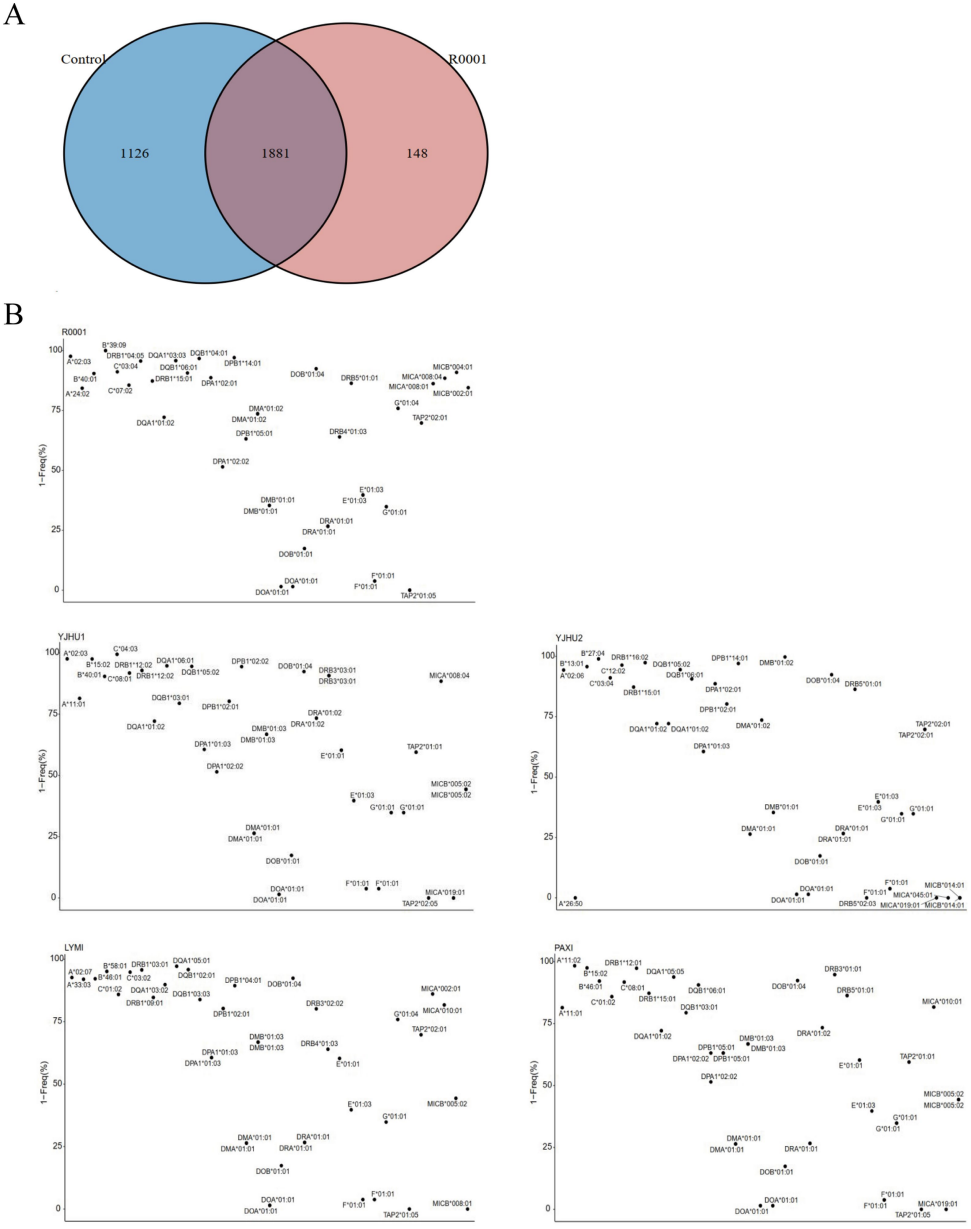
**(A)** Venn diagram of the variation site differences between the control sample and case sample. **(B)** The possibility that alarelin acetate-induced liver injury is related to *HLA* genes.

In the study, we analyzed one participant who developed acute liver injury (case) and four unaffected participants (controls) (Table 3). To investigate the genetic basis of the hepatotoxicity, we performed comprehensive PGx and *HLA* genotyping (Figure 1A). The pharmacogenomic analysis of 4,343 loci across all five samples revealed 2,044 variants in the index case (R001) (Supplementary file 1), including 18 high-frequency variants in the Chinese population (Supplementary file 2). The control variant counts were 1,986 (YJHU1), 2,056 (YJHU2), 1,981 (LYMI), and 2,018 (PAXI) (Figure 1B; Supplementary file 1). Figure 1C displays the distribution of the low-frequency variants (MAF < 5%). The comparative analysis identified 148 unique loci in R001 compared to the controls (Figure 2A; Supplementary file 3). The haplotype analysis of 10 pharmacogenes influencing drug metabolism (*CYP2B6*, *CYP2C19*, *CYP2C9*, *CYP2D6*, *CYP3A4*, *CYP3A5*, *NUDT15*, *SLCO1B1*, *TPMT*, and *UGT1A1*) revealed that R001 carried a NUDT15 *3/*3 diplotype, indicating a slow metabolizer status (Table 4).

**Table 3:** Sample information is shown in the following table.

Sample	Group
R001	Case
YJHU1	Control
YJHU2	Control
LYMI	Control
PAXI	Control

**Table 4:** Genes for drug-metabolizing enzymes.

Gene	Reference base (s)	Result
*CYP2B6*	*1/*1	*1/*1 (Normal metabolism)
*CYP2C19*	*38/*38	*38/*38 (Normal metabolism)
*CYP2C9*	*1/*1	*1/*1 (Normal metabolism)
*CYP2D6*	*1/*1	*10/*34 (Normal metabolism)
*CYP3A4*	*1/*1	*1/*1 (Normal metabolism)
*CYP3A5*	*1/*1	*1/*3 (Normal metabolism)
*NUDT15*	*1/*1	*3/*3 (poor metabolism)
*SLCO1B1*	*1/*1	*1/*37 (Normal metabolism)
*TPMT*	*1/*1	*1/*1 (Normal metabolism)
*UGT1A1*	*1/*1	*1/*1 (Normal metabolism)

CYP, cytochrome P450 enzyme; NUDT15, nudix hydrolase 15; SLCO1B1, solute carrier organic anion transporter family member 1B1; TPMT, thiopurine S-methyltransferase; UGT1A1, UDP-glucuronosyltransferase 1A1.

High-resolution *HLA* genotyping was completed for all participants (Supplementary file 4). The case participant (R001) exhibited 40 distinct *HLA* alleles (Supplementary file 5). The analysis of rare *HLA* alleles (MAF < 5% in the Chinese population) revealed the following: R001 had HLA-A*02:03, HLA-B*39:09, HLA-DPB1*14:01, HLA-DQB1*04:01, HLA-DQA1*03:03, and HLA-DRB1*04:05. In the control group, YJHU1 had HLA-A*02:03, HLA-B*15:02, and HLA-C*04:03; YJHU2 had HLA-B*13:01, HLA-B*27:04, HLA-C*12:02, HLA-DRB1*16:02, HLA-DPB1*14:01, and HLA-DMB*01:02; LYMI had HLA-B*58:01, HLA-DRB1*03:01, and HLA-DQA1*05:01; and PAXI had HLA-A*11:02, HLA-B*15:02, and HLA-DRB1*12:01 (Figure 2B; Table 5).

**Table 5:** *HLA SNPs* with MAF < 5% in Chinese population.

Sample	*SNPs* with MAF < 5% in Chinese population
R001	B*39:09, A*02:03, DPB1*14:01, DQB1*04:01, DQA1*03:03, DRB1*04:05
T001	A*02:03, B*15:02, C*04:03
T002	B*13:01, B*27:04, C*12:02, DRB1*16:02, DPB1*14:01, DMB*01:02
T003	B*58:01, DRB1*03:01, DQA1*05:01
T004	A*11:02, B*15:02, DRB1*12:01

SNPs, single nucleotide polymorphisms; MAF, minor allele frequency.

## Discussion

As a synthetic GnRH analogue, alarelin acetate is widely used in the treatment of endometriosis and hormone-sensitive tumors. However, alarelin acetate-induced DILI is extremely rare, and the mechanism is unknown. This study is the first to report a clinical case of alarelin acetate-associated DILI with an in-depth genetic analysis. This case expands the cognitive scope of the safety of GnRH analogues, suggesting that alarelin acetate may mediate hepatotoxicity through *HLA*-restricted immune mechanisms. Our study revealed three possible key mechanisms: metabolic pathway-related gene variants (CYP-UGT pathway), a *NUDT15* slow metabolism-related phenotype, and an abnormal immune response mediated by *HLA* polymorphism. Together, these factors formed the basis for triple susceptibility in this case.

The polymorphism of drug-metabolizing enzyme genes is the core factor leading to inter-individual differences observed in drug responses. In this study, we found that the case samples carried multiple low-frequency genetic variants (MAF < 5%), including *MYC* (rs4645962 T > C), *EPHA4* (rs79551441 -> T), and *FLT1* (rs12877323 T > G). These variants may increase liver sensitivity to injury by altering pharmacokinetic profiles. Notably, *MARK3* (rs5811102 -> T), *EPHA4* (rs79551441 -> T), and *ABCG2* (rs2725271 C > T) belonged to the 532 high-frequency variant loci identified in the Chinese population (16). These results indicate that ethnic-specific genetic backgrounds may play an important role in the occurrence of DILI.

A number of studies have confirmed that variants in the genes of drug transporters and metabolic enzymes are closely related to the risk of DILI. Several loci detected in this case have been previously reported to be associated with the occurrence of DILI: *ABCB1* (rs2032582 A > C) affects the hepatobiliary transport of multiple drugs (17), *CYP2C8* (rs11572078 -> A) and *CYP2E1* (rs2070676 -> C) are involved in drug oxidative metabolism (18), and *IL6* (rs2069840 C > G) regulates the inflammatory response (19). In contrast, multiple variants in the *UGT1A9* cluster (rs11568319 -> G, rs12052787 -> T, and others) affect glucuronic acid binding, a key detoxification pathway in phase II drug metabolism (20). The synergistic variation of these metabolic pathway genes may significantly reduce the liver’s ability to process drugs and their active metabolites, leading to the accumulation of toxic substances.

The presence of the NUDT15*3/*3 polymorphism (rs116855232) in this case requires further discussion. NUDT15, as a nucleotide pyrohydrolase, plays a key role in the metabolism of thiopurines, such as azathioprine (21). The hypometabolic phenotype of this gene is more common in Asian populations and leads to abnormal accumulation of the active metabolite 6-thioguanine nucleotide, increasing the risk of myelosuppression (22). Notably, the NUDT15*3/*3 genotype was found to be associated with azathioprine-induced liver injury (23). However, alarelin acetate is a peptide drug whose metabolism is theoretically independent of the *NUDT15* pathway. Our findings provide new insights into the role of *NUDT15* in the hepatotoxicity of non-thiopurines.

The polymorphism of the *HLA* system plays a decisive role in the drug-specific immune response (24–27). This study found that the patient carried multiple *HLA* alleles associated with adverse drug reactions, forming a unique immune susceptibility background (Table 6). These alleles show clear patterns associated with different organ damage, especially in the skin and liver. The skin, another common target organ, shows different characteristics of the *HLA* association. HLA-A*24:02, HLA-B*40:01, HLA-DPA1*02:02, and HLA-DRB1*04:05 carried by this patient are associated with severe skin reactions, such as antipsychotic-induced drug reaction with eosinophilia and systemic symptoms (DRESS) and Stevens–Johnson syndrome (SJS) (28–31). HLA-DQA1*03:03 and HLA-DQB1*04:01 have also been reported to be associated with drug-induced cutaneous adverse reactions (32). The liver, a major metabolic organ, exhibits significant gene-drug specificity in *HLA*-related drug-induced damage. For example, liver injury caused by amoxicillin-clavulanic acid is associated with the HLA-DRB1*15:01 and HLA-DQB1*06:01 haplotypes (33, 34). Liver injury caused by antituberculosis drugs is associated with the HLA-DQA1*01:02 haplotype (35). In addition, HLA-B*39:09 is related to clozapine-induced agranulocytosis (36, 37). HLA-DPA1*02:01 is related to a negative antibody after measles vaccination (38). By altering the structure of the antigen-binding groove, these alleles affect the recognition efficiency of the drug–antigen complex with the T-cell receptor, resulting in pathological immune activation.

**Table 6:** *HLA* genotyping of R001 and ADR.

HLA type	Drug	ADR
HLA-A*24:02	Phenytoin, Lamotrigine, Methazolamide	DRESS/SJS/TEN
HLA-B*40:01	Carbamazepine	DRESS
HLA-DRB1*04:05	Methazolamide	SJS/TEN
HLA-DPA1*02:02	Carbamazepine	MPE
HLA-DQA1*03:03	Lamotrigine	Cutaneous adverse drug reactions
HLA-DQB1*04:01	Lamotrigine	Cutaneous adverse drug reactions
HLA-DRB1*15:01	Amoxicillin/clavulanic acid	Hepatitis
HLA-DQB1*06:01	Amoxicillin/clavulanic acid	Hepatitis
HLA-DQA1*01:02	Antituberculosis drugs	Hepatotoxicity
HLA-B*39:09	Clozapine	Agranulocytosis
HLA-DPA1*02:01	Measles vaccine	Negative antibody

DRESS, drug reaction with eosinophilia and systemic symptoms complex; SJS, Stevens-Johnson syndrome; TEN, toxic epidermal necrolysis; MPE, maculopapular exanthema.

Although this study provides important genetic insights into alarelin acetate-associated DILI, there are still some limitations: (a) sample size limitation: It is difficult to distinguish pathogenic mutations from benign polymorphisms in single-case reports, and it is necessary to expand the sample size in order to verify key genetic markers. (b) Lack of functional validation: the actual ability of *HLA* molecules to present the alarelin acetate antigen or elicit a T-cell response was not confirmed. (c) Insufficient integration of multi-omics data: There was a lack of synergistic analysis of epigenetic regulation, transcriptomic data, and proteomic information. (d) Insufficient population diversity: The study was based on an Asian population, and the results may not apply to other ethnic groups.

## Conclusion

In the study, we incidentally discovered and reported that alarelin acetate may induce the occurrence of DILI. We were the first to elucidate the possible causes of alarelin acetate-associated DILI by genetic analysis; however, larger clinical studies are needed to further confirm this hypothesis. In addition, this pharmacovigilance report implies the value of preemptive PGx/*HLA* screening in clinical trials.

## Data Availability

The original contributions presented in the study are included in the article/Supplementary material, further inquiries can be directed to the corresponding author.

## Supplementary material

SUPPLEMENTARY FILE 1 The pharmacogenomics results of 4,343 locus in 5 samples was assessed, 2,044 (R001), 1,986 (YJHU1), 2,056 (YJHU2), 1,981 (LYMI) and 2,018 (PAXI) pharmacogenomic mutations were detected.

SUPPLEMENTARY FILE 2 18 high-frequency genes in the Chinese population were found in R001.

SUPPLEMENTARY FILE 3 148 unique loci were found in R001.

SUPPLEMENTARY FILE 4 The genotyping of 22 *HLA* genes in 5 samples.

SUPPLEMENTARY FILE 5 Forty *HLA* alleles were detected in R001 by *HLA* genotyping.

## Glossary

GnRH: gonadotropin-releasing hormone
SNPs: single-nucleotide polymorphisms
DILI: drug-induced liver injury
HLA: human leukocyte antigen
ALT: alanine aminotransferase
AST: aspartate aminotransferase
LDH: lactate dehydrogenase
ALP: alkaline phosphatase
GGTγ: Glutamyltransferase
TBil: total bilirubin
PGx: pharmacogenomic
CYP450: cytochrome P450 proteins
UGTs: UDP-glucuronosyltransferases
GST: glutathione S-transferases
NAT: N-acetyltransferase
MAF: minor allele frequency
RUCAM: Roussel Uclaf Causality Assessment Method
NUDT15: NUDIX-type 15
DRESS: drug reaction with eosinophilia and systemic symptoms
SJS: Stevens–Johnson syndrome
TEN: toxic epidermal necrolysis
MPE: maculopapular exanthema
