# Supplementary material for: Pharmacogenomic analysis of alarelin acetate-induced hepatotoxicity: a case report and literature review
Source: Front Med (Lausanne). 2025 Sep 29;12:1634101. doi: 10.3389/fmed.2025.1634101 (PMC12515876; doi:10.3389/fmed.2025.1634101)
Supplement: SUPPLEMENTARY FILE 5 — Forty HLA alleles were detected in R001 by HLA genotyping. [file Table_1.docx]

Supplementary table 1. The list of reagent kits and lot numbers used in pharmacogenomic profiling.

|  | Reagent kits | Number |
| --- | --- | --- |
| The library preparation for Pharmacogenomics analysis | the IGT® Enzyme Plus Library Prep Kit V3 | Lot No. 68142201 |
|  | the IGT® Adapter & UDI Primer 1-96 | Lot No. 6A136202 |
|  | the IGT® Pure Beads | Lot No. 6A126201 |
| The entire hybridization capture process for Pharmacogenomics analysis | the TargetSeq One® Hyb & Wash Kit v2.0 | Lot No. 69142201 |
|  | the TargetSeq One® Hyb & Wash Kit v2.0 | Lot No. 6A146201 |
|  | the TargetSeq One® Hyb & Wash Kit | Lot No. 68104201 |
|  | the TargetSeq® Eco Universal Blocking Oligo | Lot No. 68146201 |
|  | the TargetSeq® Cap Beads & Nuclease-Free Water | Lot No. 69130201 |
| The library preparation for *HLA* high-resolution genotyping | the NadPrep® EZ DNA Library Preparation Module v2 | Lot No. 2602332701 |
|  | the NadPrep® Universal Stubby Adapter Module Set E1 | Lot No. 3252320901 |
| The entire hybridization capture process for *HLA* high-resolution genotyping | the NadPrep® Hybrid Capture Reagent | Lot No. 5102325501 |
|  | the NadPrep® NanoBlockers | Lot No. 11022322601 |
